# Mussel-Inspired Fabrication of Konjac Glucomannan/Poly (Lactic Acid) Cryogels with Enhanced Thermal and Mechanical Properties

**DOI:** 10.3390/ijms18122714

**Published:** 2017-12-16

**Authors:** Lin Wang, Yi Yuan, Ruo-Jun Mu, Jingni Gong, Yongsheng Ni, Xin Hong, Jie Pang, Chunhua Wu

**Affiliations:** College of Food Science, Fujian Agriculture and Forestry University, Fuzhou 350002, China; brandonwjl53667@163.com (L.W.); yuanjohnay1233@163.com (Y.Y.); muruojun@gmail.com (R.-J.M.); ggjjmna@gmail.com (J.G.); dragonyongsheng@163.com (Y.N.); 1160912007@fafu.edu.cn (X.H.)

**Keywords:** nanofibers cryogels, konjac glucomannan, polydopamine, poly (lactic acid), mechanical properties

## Abstract

Three-dimensional nanofibers cryogels (NFCs) with both thermally-tolerant and mechanically-robust properties have potential for wide application in biomedical or food areas; however, creating such NFCs has proven to be extremely challenging. In this study, konjac glucomannan (KGM)/poly (lactic acid) (PLA)-based novel NFCs were prepared by the incorporation of the mussel-inspired protein polydopamine (PDA) via a facile and environmentally-friendly electrospinning and freeze-shaping technique. The obtained KGM/PLA/PDA (KPP) NFCs were characterized by field emission scanning electron microscopy (FE-SEM), Fourier transform infrared spectroscopy (FTIR), X-ray diffraction (XRD), thermogravimetric analysis (TGA), differential scanning calorimetry (DSC) and compressive and tensile test. The results showed that the hierarchical cellular structure and physicochemical properties of KPP NFCs were dependent on the incorporation of PDA content. Moreover, the strong intermolecular hydrogen bond interactions among KGM, PLA and PDA also gave KPP NFCs high thermostability and mechanically-robust properties. Thus, this study developed a simple approach to fabricate multifunctional NFCs with significant potential for biomedical or food application.

## 1. Introduction

Three-dimensional nanofibers cryogels (NFCs) are a kind of highly porous, light-weight and highly continuous solid material with many exciting properties, including low density, low dielectric permittivity, high specific surface area, as well as excellent low thermal conductivity. Owing to these merits, NFCs have broad applications in the fields of biomedicine [1,2,3], adsorbents [4,5,6], catalyst supports [7,8], biosensors and diagnostics [9,10,11]. NFCs’ materials normally have cellular architectures [12], which are synthesized by the assembly of various nanofibers. Several cellulosic materials, including bacterial cellulose fibrils [13] and cellulose nanocrystals [14], have recently been used as building blocks for NFCs [14,15,16,17]. However, creating excellent NFCs only from natural polymers has proven to be extremely challenging due to the inherent limits of the structural diversity of these biopolymeric nanomaterials [18].

To obtain homogeneous biopolymer-based NFCs materials, several processing techniques, such as phase separation [19], self-assembly process [20] and electrospinning [21], have been developed [22]. Among them, electrospinning is a powerful tool to produce fiber mats from different materials [23,24]. Additionally, its products, the electrospun nanofibers, were found to combine the properties of robust mechanical strength [25,26], low density [27], high degree of flexibility [28], extremely high aspect ratio [29] and ease of scalable synthesis from various materials [30] (polymer, ceramic, metal, carbon, and so on). Thus, such nanofibers hold great potential as an exceptional nanoscale building block for constructing macroscopic NFCs. For instance, Adlhart et al. processed nanofibers into ultra-light sponges or aerogels, and these sponges or aerogels also exhibit a hierarchical porous structure [31] 

Polysaccharides have received much attention in the biomedical and food area over the past few years. Among these materials, konjac glucomannan (KGM) is recognized as a preferred natural polysaccharide [32] because of its nontoxicity [33], excellent biodegradability [34,35] and biocompatibility [36] and good film-forming ability [33]. KGM gels and films are thus promising candidates for wound dressing and food packaging [37,38]. However, the wide usage of KGM in industry has remained restricted in recent years. The electrospinning of KGM is extremely difficult and challenging due to its high molecular weight, viscosity and three-dimensional networks of hydrogen bonds. Recently, Yuan et al. used galactoglucomannan (GGM) to prepare KGM/GGM nanofilm, which enhanced the thermal stability of the KGM nanofiber membrane and solved the problem of the high water solubility of KGM [39].

Poly (lactic acid) (PLA) is a good electrospinning material [40,41] due to its strong mechanical strength [42] and good fiber-forming ability [43]. Electrospun blending of PLA with natural polymer has been applied in scaffolds [44], biodegradable films [45] and nanofibers [46]. PDA shows a special adhesive ability in the presence of residual catechol groups [47]. Yan et al. [46] and Kasemset et al. [48] prepared a nanofiber membrane with polydopamine (PDA), which can be used for adsorption and ultrafiltration. For hydrophilic materials, KGM can form hydrogen-bonding interactions with PDA, which belongs to a class of catecholamines. It was able to self-polymerize under basic reaction conditions and form a PDA layer onto almost all types of substrates. Although blending KGM into PLA can combine the superior properties of both PLA and KGM, no report on electrospun KGM/PLA nanofibers and KGM/PLA/PDA cryogels have been published.

**Scheme 1:**
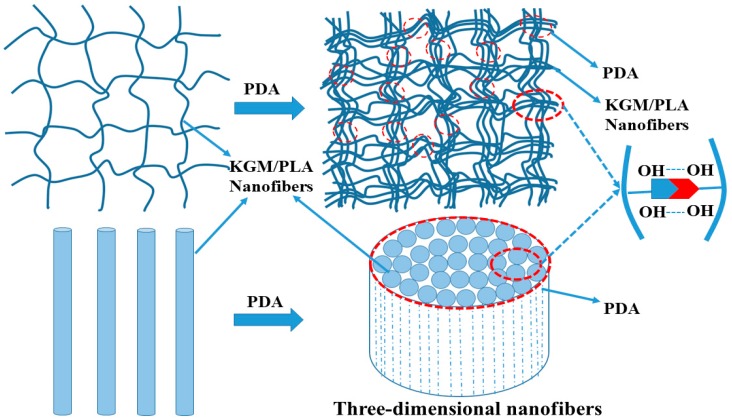
The mechanism diagram and hydrogen-bond interaction (red dotted circle) of KGM/PLA nanofibers and PDA.

Inspired by natural mussel chemistry, we proposed a simple approach to prepare KGM/PLA NFCs, with a hierarchical cellular structure and high thermally-tolerant and mechanically-robust properties, via a facile and environmentally-friendly electrospinning and freeze-shaping technique. In our NFC system, the electrospun KGM/PLA nanofiber was chosen as a primary skeleton, and PDA was used as a cross-linker(see Scheme 1). Moreover, FE-SEM, XRD, FTIR and TGA were utilized to evaluate the structure and properties of the KGM/PLA/PDA (KPP) NFC materials we developed. The novel KPP NFCs could be promising carriers for biomedical or food systems. 

## 2. Results and Discussion

### 2.1. Morphology of the Electrospun KGM/PLA Nanofibers

**Figure 1:**
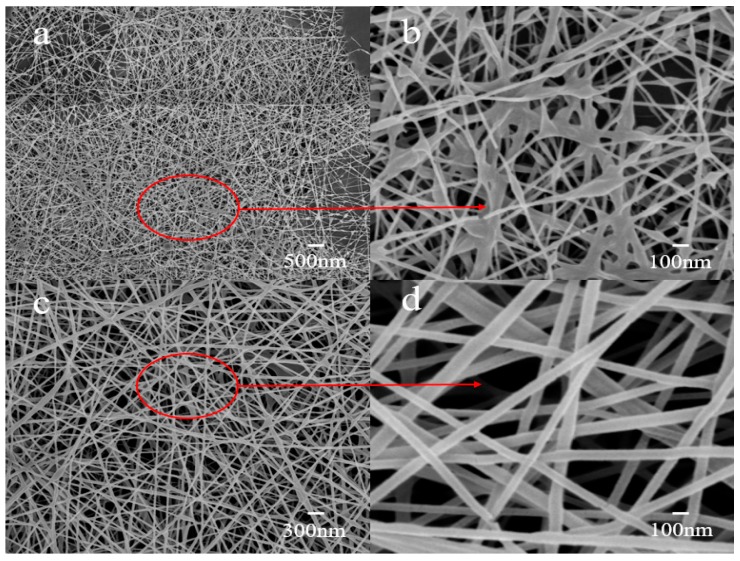
The morphology image of the electrospun KGM nanofibers (**a**,**b**) and KGM/PLA nanofibers (**c**,**d**).

Nanofiber membranes’ surface morphology showed the basic structure. The surface structures of the KGM and KGM/PLA nanofiber membranes were observed through SEM. Figure 1a,b shows that the 1.2% pure KGM nanofiber membrane was heavily tangled, with uneven fiber diameters. This observation could be explained by the structural collapse of the KGM nanofiber membrane when it rapidly absorbed water. However, in Figure 1c and d the appearance of the prepared KGM/PLA nanofiber membrane was very smooth and delicate, with uniform diameter and few entanglements. The observed diameter of nanofibers was approximately 100–130 nm. Moreover, the internal structure of the nanofiber membrane was netlike in appearance, with a randomized distribution and numerous small gaps. This type of structure provides better properties and a large specific surface area for the membrane. Furthermore, the addition of PLA can effectively improve the structure of KGM/PLA nanofiber membranes because of the interaction and physical entanglement between KGM and PLA.

### 2.2. Formation of Hierarchical Cellular-Structured KPP NFCs and SEM of the Cryogels

**Figure 2:**
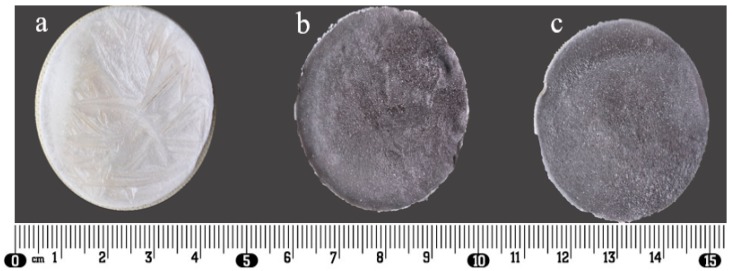
The images of (**a**) KPPA0; (**b**) KPPA2 and (**c**) KPPA4 cryogels.

**Figure 3:**
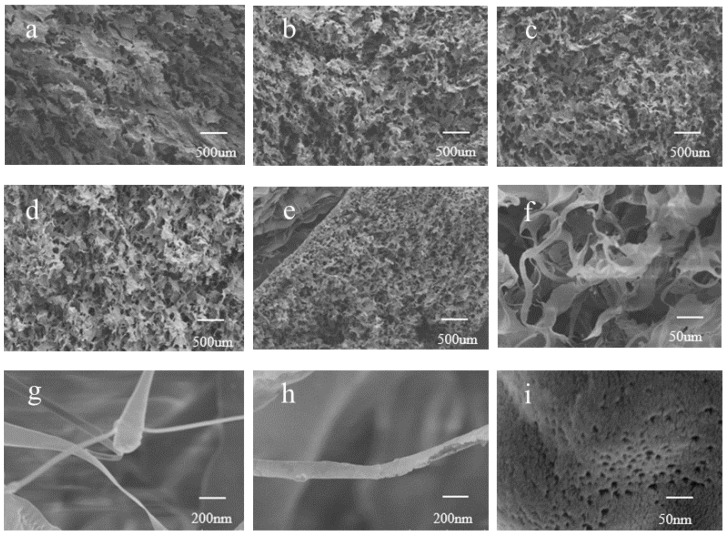
The image of KPP NFCs and the FESEM images of (**a**) KPPA0; (**b**) KPPA1; (**c**) KPPA2; (**d**) KPPA3; (**e**) KPPA4; (**f**–**h**) fibers of KPPA4 and (**i**) the pore structure of KPPA4.

KGM/PLA nanofibers were selected as the major precursors to construct the fibrous networks, and polydopamine (PDA) was blended into KGM/PLA nanofibers as a novel in situ crosslinking agent, which were introduced into cryogels to enhance their structural stability. Figure 2 and Figure 3 demonstrate the scalable fabrication of hierarchical cellular-structured fiber NFCs through the combination of electrospinning and fibrous freeze-shaping methods. The surface and microcosmic morphology of KPP NFCs are shown in the Figure 2 and Figure 3. A porous structure with many pores and a network structure were clearly observed for the KPPA0 (KGM/PLA cryogel) (Figure 3a). To facilitate a more compact structure, the addition of PDA showed an obvious compacting effect on the microcosmic morphology of KPPA1 (Figure 3b–e). The KPPA2, KPPA3 and KPPA4 cryogels exhibited many pores and a network structure, which might be attributed to the synergy interaction and physical entanglement among KGM, PLA and PDA. The fibers can be clearly observed (Figure 3f–h), since more pores formed on the surface after increasing the volume ratio of KPPA4 cryogels. The surface of KPP NFCs exhibited not only a hierarchical cellular structure, but also the most compact and pore-rich structure (Figure 3b–e). It is obvious that the nano-pores are distributed uniformly on the internal part of the KPPA3 and KPPA4 cryogels fibers. The PDA content has an impact on the fiber preparation and pore formation (Figure 3i). In addition, the average fiber diameter was not influenced in the range from about 150–200 nm. Calculated from the porosity formula, the porosity of KPPA4 cryogels is in the range of 30–35%, and it can be seen from the pore electron micrographs (Figure 3i). The pore diameter of KPPA4 cryogels is less than 50 nm.

### 2.3. FTIR Spectral Analysis

**Figure 4:**
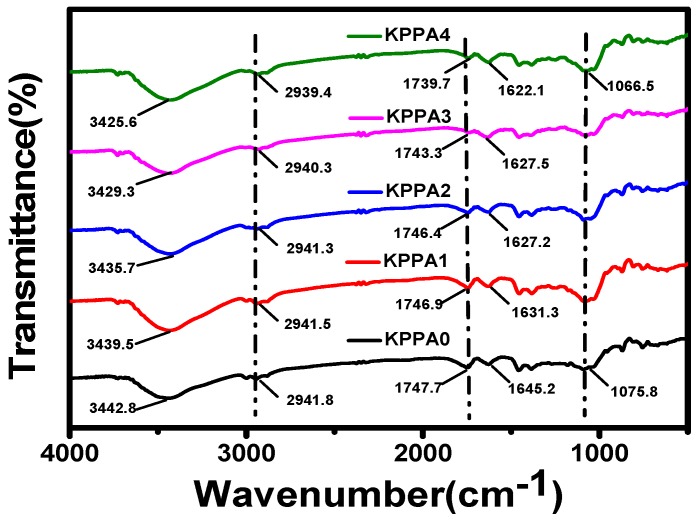
FTIR spectra of the KPPA0, KPPA1, KPPA2, KPPA3 and KPPA4 cryogels.

The FTIR spectra of KPP NFCs are shown in Figure 4. All the spectra showed that O–H and N−H stretching vibration occurs between 3600 and 3000 cm^−1^ [49]; the peak at 2939 cm^−1^ is assigned to the stretching vibrations of methylene groups; the peak at 1739 cm^−1^ is assigned to the stretching of the C=O of the carbonyl of acetyl groups and the C–O of the intermolecular hydroxyl groups. The sharpest band of the KPPA4 spectrum is 1622.1 cm^−1^, which corresponds to the carbonyl stretching vibration. However, for the KPPA0, KPPA1, KPPA2, KPPA3 and KPPA4 cryogels, the stretching vibrations of –OH groups were generated at 3442.8, 3439.5, 3435.7, 3429.3 and 3425.6 cm^−1^, respectively; while the bending vibrations of –C–H groups occurred at 1645.2, 1631.3, 1627.5,1627.2 and 1622.1 cm^−1^ [50], respectively. The shift of these absorption peaks indicated that the intermolecular hydrogen bonding between PDA and KGM/PLA molecular chains was formed in the cryogels.

The stretching vibration of the carboxyl group was generated at 1739.7, 1743.3, 1746.4, 1746.9 and 1747.7 cm^−1^, in KPPA0, KPPA1, KPPA2, KPPA3 and KPPA4 cryogels, respectively (Figure 4). The concentration of the PDA in the KPP NFCs has a great effect on the intensity of the FTIR spectrum, and with the increase of the amount of PDA in the KPP NFCs, the characteristic absorption bands of the PDA also increased. The absorption peaks at 1739.7, 1743.3, 1746.4 and 1746.9 cm^−1^ became weaker in KPP NFCs, partly demonstrating the physical/chemical combinations between PDA and KGM chains.

### 2.4. X-ray Diffraction Analysis

**Figure 5:**
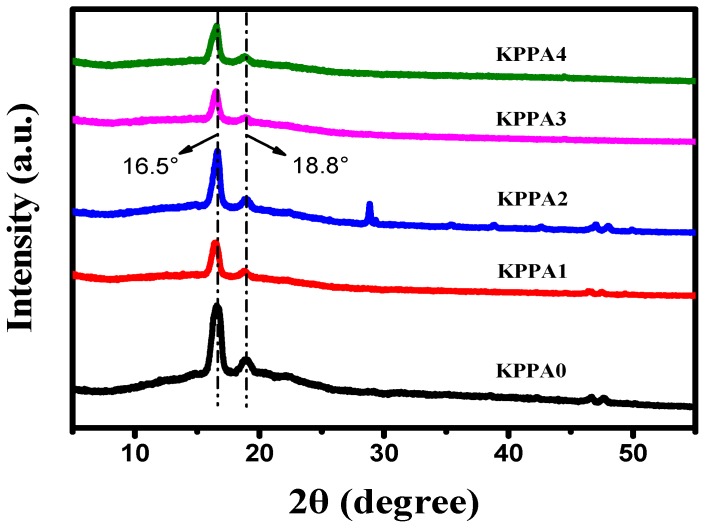
X-ray diffraction patterns of the KPPA0, KPPA1, KPPA2, KPPA3 and KPPA4 cryogels.

For further investigating the changes in crystal structure [51,52], the XRD patterns of the KPPA0, KPPA1, KPPA2, KPPA3 and KPPA4 cryogels were compared. As shown in Figure 5, all samples exhibited a sharp peak at around 16.5° and a small peak at around 18.8°. In comparison to KPPA0, there is a smaller characteristic peak at around 16.5° and 18.8° in the XRD patterns of KPP NFCs, which suggested that PDA was uniformly dispersed in the polymer cryogels, with fewer ordered aggregation. Nevertheless, the crystallinity index of KPPA4 cryogels was lower than that of the KPPA0, which suggested the existence of a more amorphous material.

### 2.5. Thermogravimetric Analysis

**Figure 6:**
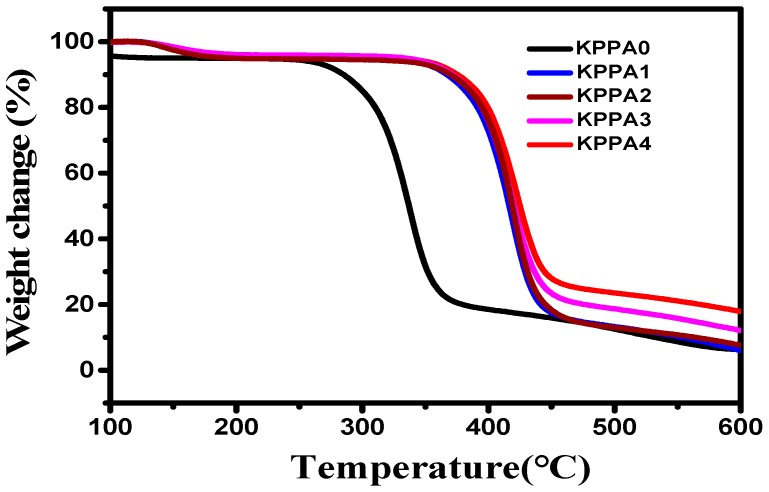
TGA curves of the KPPA0, KPPA1, KPPA2, KPPA3 and KPPA4 cryogels.

The thermal stability of the KPPA0, KPPA1, KPPA2, KPPA3 and KPPA4 was studied by the TGA technique (Figure 6). The KPPA0 shows two main exothermic peaks. This might be caused by two reasons, the hydrophilicity of KGM; and the weight loss during the evaporation of residual water from the non-bound water or the aerosol surface [53]. The former (200–320 °C) is mainly due to KGM intermolecular water discharge, hydrogen bond breakage, decomposition of the sugar ring in the molecule or molecular side chain removal by the degradation effect. The latter (320–400 °C) is mainly due to further decomposition of the molecular chain caused by the degradation effect [54].

From the TGA curve, the thermal weight loss curves of KPP NFCs are above that of the KGM/PLA cryogels in the tested temperature range. This finding suggested that the thermal stability of KPP NFCs might be better than that of KPPA0. The high amount of oxygen-containing groups in KGM, PLA and PDA molecules, capable of forming hydrogen bonds, may have influenced the TGA curve. Meanwhile, physical entanglements may have occurred between the KGM molecules and the PDA attached to the molecular chains of KGM. Furthermore, the hydrogen bonding interaction of KGM/PLA and PDA molecules in the cryogels may also improve the thermal stability of cryogels. In contrast, the KPPA0 cryogels have a poor thermal stability, the strongest peak of which is centered at 327.8 °C, much lower than that of the others.

The thermal decomposition of KGM is extended over a wider temperature range (200–600 °C) according to the interaction of PLA or PDA on the pyrolysis of cryogels. Compared to the KPPA0 cryogels, the KPP NFCs displayed more superior thermal stability. The strongest peaks of the KPP NFCs are located at 387.7, 388.9, 390.2 and 391.6 °C, much higher than that of the KPPA0 cryogels (327.8 °C). Thus, the maximum degradation rate of the KPP NFCs might have shifted to a higher temperature.

**Figure 7:**
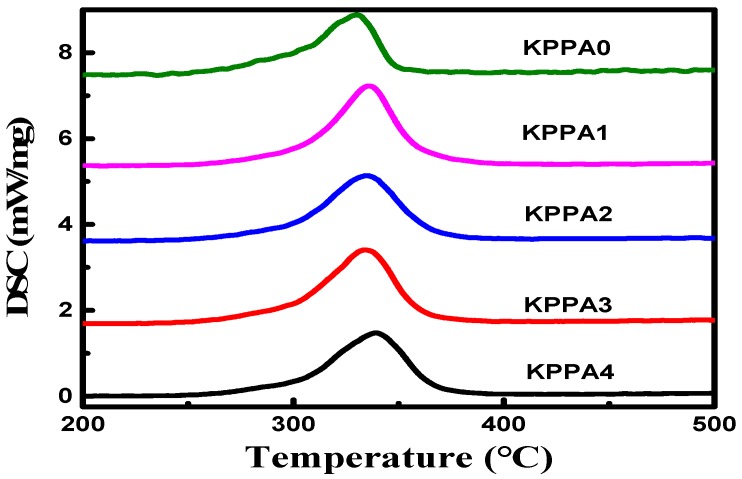
The DSC curve of the KPPA0, KPPA1, KPPA2, KPPA3 and KPPA4 cryogels.

The differential scanning calorimetry (DSC) thermograms of KPPA0, KPPA1, KPPA2, KPPA3 and KPPA4 cryogels are presented in Figure 7. The addition of PDA resulted in an increase in the exothermic peak. This may be attributed to the thermal degradation of KGM in KPP NFCs. Values obtained from the determination of the exothermic peak area showed an increasing trend as the amount of PDA increased. The increase is an indication of enhanced thermal stability, due to the interaction between the molecular chains of KGM and PDA during the formation of the KPP NFCs. A high amount of oxygen-containing groups, capable of forming hydrogen bonds, in KGM and PDA molecules may have influenced the interaction. Thus, the results of DSC are consistent with TGA analysis, since PDA can significantly increase the thermal stability of the KPP NFCs. 

### 2.6. Mechanical Properties of Cryogels

**Figure 8:**
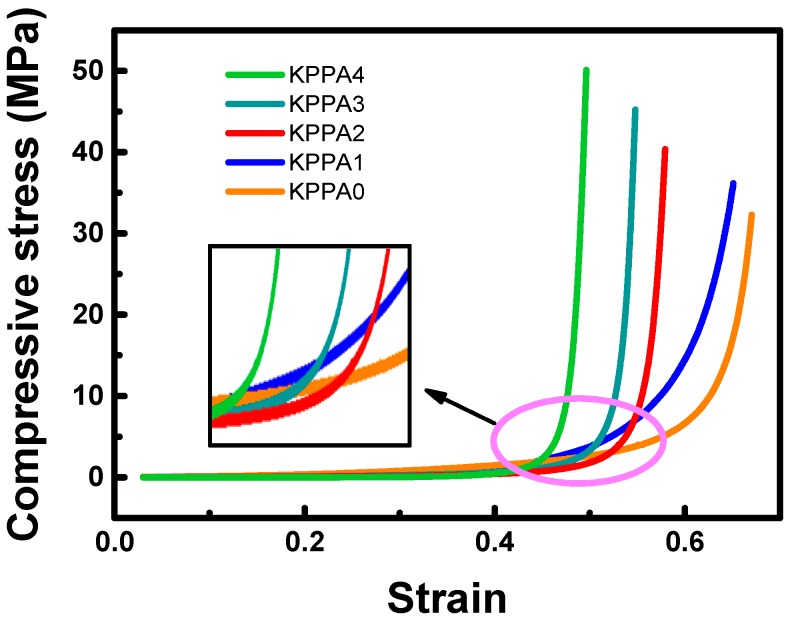
Compressive stress of the KPPA0, KPPA1, KPPA2, KPPA3 and KPPA4 cryogels.

**Figure 9:**
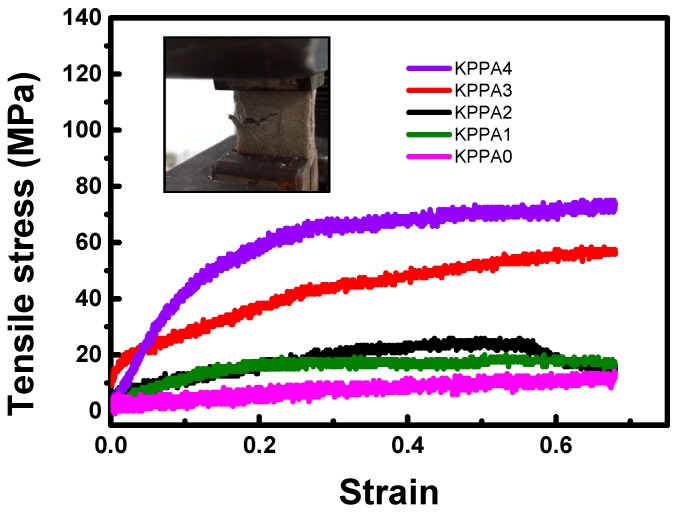
Tensile stress of the KPPA0, KPPA1, KPPA2, KPPA3 and KPPA4 cryogels.

The mechanical properties of a composite can reflect its homogeneity state and the interfacial interactions between the constituents of the composite [55]. Typical stress-strain performance of the KPP NFCs with different PDA are shown in Figure 8 and Figure 9.

The compressive strength of the obtained cryogels was increased with the increase of PDA content (Figure 8). The higher compressive strength for KPP NFCs might be attributed to the reduced formation of increased density after PDA doping. The decrease of the average pore size was attributed to the interaction between PDA and KGM, which resulted in an increased density of cryogels, thereby significantly improving the mechanical properties of the cryogels. The compressive stress increased from 2.0 MPa for KPPA0 cryogel to 50 MPa for KPPA4 cryogels, which was enhanced by nearly 25 times. The zoomed-in initial stress-strain curves are also shown in the inset of Figure 8. The compressive stress-strain curves at higher strain rates are generally higher than those at lower strain rates, indicating a remarkable strain rate strengthening effect of KPP NFCs within these strain rates. In addition, KPP4 cryogels show stiffened behavior at high strain rates in comparison to the KPP0 cryogels. 

The values of Young’s modulus (E) for KPP0 and KPP4 are 18.2 and 35 MPa, respectively; and the ultimate compressive strength of these two materials are 32.2 and 50.1 MPa, respectively. The Young’s modulus of KPP4 cryogels is about 48% higher than that of KPP0 cryogel. PDA in the cryogel not only strengthened the links between nanofibers, but also increased the density of cryogels, so that its viscosity does contribute much to the strength of the material. Thus, the KPP0 cryogel is weaker than the KPP4 cryogels. The ultimate compressive strength of KPP4 cryogels increased by 36%, compared to the KPP0 cryogel. 

Similarly, the tensile strength and the yield of cryogels increased with the increase of PDA content, as shown in Figure 9. KPPA4 cryogels have good tensile strength. At the initial stage of the stretching, the curve exhibits a distinct linear character [56]. As the stress increases, the curve starts to appear to have a more nonlinear shape [57]. When the stress reaches its maximum, the specimen starts to crack. Later, the crack starts to expand in the thickness direction under the continuous action of the tensile loading. The test of compressive and tensile strength indicated that KPP NFCs have strong hydrogen-bond interaction, while KPP0 cryogel has low mechanical properties, and when the nanofibers are cross-linked with polydopamine, the KGM/PLA nanofibers will become three-dimensional nanofibers, which will give the KPP NFCs excellent mechanical properties due to the strong hydrogen-bond interaction.

## 3. Materials and Methods 

### 3.1. Materials

Konjac glucomannan (KGM) (purity of 95%, viscosity: 1% solution, 30 °C, ≥35,000 MPa) was supplied by San Ai Konjac Food Co., Ltd. (Yunnan, China). Dopamine and poly (lactic acid) were purchased from Aladdin reagent Co., Ltd. (Shanghai, China). Dichloromethane (DCM) (boiling point: 39.8 °C) and tetrahydrofuran (THF) (boiling point: 66 °C) were obtained from Tianjin Fengchaua Chemical Reagent Co., Ltd. Other analytical-grade chemical reagents were purchased from Sino pharm Group Chemical Reagent Co., Ltd. (Shanghai, China). 

### 3.2. Synthesis of PDA

The PDA was obtained by the dopamine oxidation self-polymerization reaction according to the published protocol with modifications [58]. Briefly, dopamine (2.0 mg/mL) was dissolved in 10 mL of deionized water and stirred for 10 min. The mixed solution (360 mL of deionized water, 160 mL of absolute ethanol and 80 mL ammonia) was stirred for 30 min. The mixture was slowly supplied with 10 mL of dopamine solution. After the reaction was carried out at room temperature for 32 h, the solution was centrifuged at 10,000 rpm for 10 min. The obtained precipitate was polydopamine, and it was lyophilized and stored.

### 3.3. Formation of KGM/PLA Nanofiber Membrane

Firstly, PLA was dissolved in a binary solvent system of THF and DCM with the volume ratio of 2/1. The solutions were stirred at 45 °C to dissolve the polymer. For KGM/PLA nanofibers, the precursor solution was prepared by dissolving KGM in deionized water at the concentration of 1.2%, and then, the two substances were mixed homogeneously by an electric mixer, to obtain the spinning solutions. The electrospinning was performed using a SS-2535H electrospinning apparatus (Ucalery technology Co., Beijing, China) with an applied voltage of 15.0 kV. The solution was loaded into a syringe capped with a 6-G metal needle with a controllable feed rate of 1 mL per hour. A high voltage of 15 kV was applied to the needle tip, resulting in the generation of a continuous jetting stream [18]. The positive electrode of the electrospinning apparatus was connected to the needle with an inner diameter of 0.6 mm, and the collection plate was connected to the negative electrode [59]. The jet velocity, spinning voltage, spinning temperature, spinning distance and spinning time were set to 1.5 mm/min, 16 kV, 50 °C, 13 cm and 120 min, respectively. As a result, the KGM/PLA solution was spun out as a polymer nanofiber and collected on a metal plate. The nanofiber membrane was then collected from the collector after 2 h of spinning.

### 3.4. Preparation of KPP NFCs

**Figure 10:**
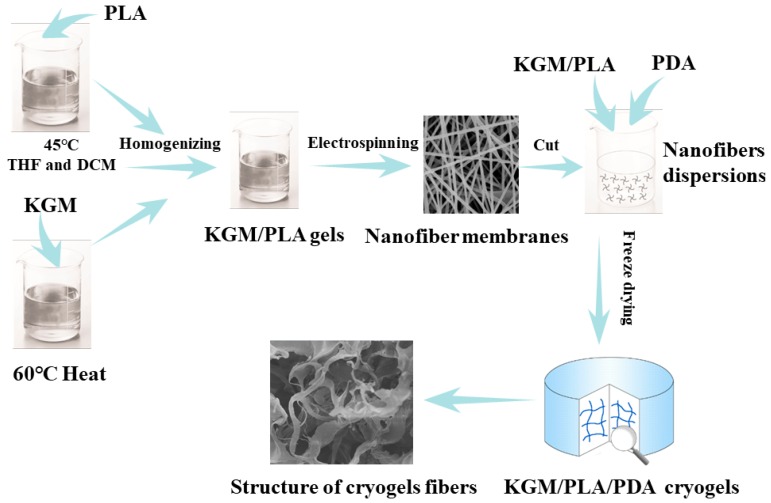
Synthesis scheme of biodegradable nanofiber-assembled NFCs based on konjac glucomannan (KGM)/polydopamine (PDA)/poly (lactic acid).

The synthesis scheme of biodegradable nanofiber-assembled cryogels is shown in the Figure 10. For the synthesis of KPP NFCs, 1.0 g of KGM/PLA nanofiber membranes was cut into 1 × 1 cm^2^ pieces and dispersed in 99 g water by homogenizing the mixture for 20 min at 10,000 rpm using a DW-S20-3000 homogenizer, yielding a uniform nanofiber dispersion. Ten, 20, 30 and 40 mL PDA (2.0 mg/mL) were then accurately measured into separate beakers, and the solutions were diluted with deionized water to a total volume of 100 mL, after which the KGM-PLA nanofibers’ dispersions were homogenized in water to form evenly-dispersed nanofiber dispersions. After being frozen at −28 °C for 12 h, the chemicals were then freeze dried to become KPPA, and they were denoted as KPPA0, KPPA1, KPPA2, KPPA3 and KPPA4, respectively.

### 3.5. Characterizations of KPP NFCs

Fourier transform infrared (FTIR) spectra of the cryogels were analyzed in KBr tablets by using a Bruker vertex 70 (Bruker, Karlsruhe, Germany) spectrometer in the range of 4000~400 cm^−1^. The compressive stress and tensile stress of cryogels were analyzed by the a rotational rheometer Malvern kinexux (Malvern Instruments Ltd., Malvern, UK). Thermogravimetric measurements were carried out with a SDTQ600 Thermo-gravimetric analyses (TGA) apparatus (TA Instruments, Lukens Drive, New Castle, DE, USA), by heating the samples at a rate of 10 °C min^−1^ from room temperature to 600 °C. Differential scanning calorimetry (DSC) was measured through a DSC200F3 (Zetzsch, Serb, Germany) with the temperature increasing from 25–500 °C at a heating rate of 10 °C/min in a nitrogen atmosphere, and the flow rate was set at 25 mL/min. The micromorphology of the cryogels was investigated by Quanta 200 high-resolution scanning electron microscope low vacuum mode (FEI-Japan, Tokyo, Japan). All the samples were coated with gold and then scanned on an accelerating voltage of 15 kV. X-ray diffraction patterns were recorded in the range of 2θ = 2–55° on the rigaku d/max 2500 X-ray Diffractometer (Rigaku, Tokyo, Japan), with Cu radiation at a scan rate (2 h) of 4 min^−1^ at 40 kV. To quantitatively evaluate the porosity of the KGM-PLA nanoporous fiber, the porosity of each sample was measured, and the average fiber diameter, pore diameter and porosity of each cryogel were measured by the image analysis software (Image Pro Plus 6.0, Georgia Avenue, Silver Spring, MD, USA) and then calculated using the equation:

(1) $$Porosity\;\left(\%\right)=\frac{\sum S_{pore}}{S_{fiber}}\times 100\%$$

The stress, strain, ultimate tensile strength and Young’s modulus were calculated according to the strains measured, as follows [60,61]:

(2) $$\sigma \left(t\right)=E\left(\frac{A_{b}}{A_{s}}\right)\varepsilon_{T}\left(t\right)$$

(3) $$\varepsilon \left(t\right)=-\left(\frac{2C_{0}}{L}\right)\int_{0}^{t}\varepsilon_{R}\left(t\right)dt$$

(4) $$E=\left(\frac{F\;L}{A\;\Delta L}\right)$$

where ε*_T_*(*t*) and ε*_R_*(*t*) denote the amplitudes of the transmitted and reflected strain pulses. *E*, *A*_0_ and *C*_0_ denote the Young’s modulus, cross-sectional area and longitudinal wave speed of the bars, and *Ar* and *L* are the cross-sectional area and length of the specimen, respectively. σ(*t*) and ε(*t*) are the stress and strain; *F* and Δ*L* are compression pressure and relative length.

## 4. Conclusions

In summary, the nanofiber-assembled KPP NFCs with different PDA contents have been prepared via a facile and environmentally-friendly electrospinning and freeze drying technique. The microstructures and morphologies of the KPP NFCs were characterized. The results demonstrate that there is a strong hydrogen-bond interaction between KGM, PLA and PDA. The surface of KPP NFCs exhibits a porous structure, and there are numerous stable nanofibers in the cryogels. The SEM images showed that the increased PDA obviously changed the macrostructures of KPP NFCs, which were in agreement with FTIR spectra, DSC, XRD and TGA results. These results supported the hypothesis that the properties of KPP0 cryogel are enhanced by PDA. Furthermore, with the increasing of PDA content, the mechanical properties and thermal stability of KPP NFCs also enhanced.
